# Supplementary material for: Polymorphisms in the vitamin D receptor gene are associated with reduced rate of sputum culture conversion in multidrug-resistant tuberculosis patients in South Africa
Source: PLoS One. 2017 Jul 10;12(7):e0180916. doi: 10.1371/journal.pone.0180916 (PMC5507304; doi:10.1371/journal.pone.0180916)
Supplement: S1 Table — (DOCX) [file pone.0180916.s001.docx]

**S1 Table.** Vitamin D receptor gene single nucleotide polymorphisms not significantly associated with initial time to sputum culture conversion

| **SNP** | **P-value^A^** | **SNP** | **P-value^A^** | **SNP** | **P-value^A^** |
| --- | --- | --- | --- | --- | --- |
| rs10875705 | 0.052 | rs7963776 | 0.337 | rs2525049 | 0.634 |
| rs987849 | 0.062 | rs11568820 | 0.341 | rs11574110 | 0.668 |
| rs2239185 | 0.063 | rs2239179 | 0.354 | rs7302235 | 0.671 |
| rs35609792 | 0.070 | rs11168309 | 0.359 | rs60556433 | 0.676 |
| rs10875700 | 0.079 | rs11168328 | 0.366 | rs58187695 | 0.683 |
| rs74088704 | 0.087 | rs11574070 | 0.387 | rs58426141 | 0.718 |
| rs7309452 | 0.092 | rs61919101 | 0.399 | rs4237855 | 0.725 |
| rs11168319 | 0.101 | rs6580642 | 0.401 | rs10467099 | 0.732 |
| rs74086592 | 0.104 | rs7976091 | 0.416 | rs58436504 | 0.735 |
| rs7974905 | 0.110 | rs739837 | 0.419 | rs3819545 | 0.737 |
| rs2246001 | 0.118 | rs12313208 | 0.421 | rs12308082 | 0.740 |
| rs1544410 | 0.120 | rs12717991 | 0.425 | rs10747526 | 0.772 |
| rs4334089 | 0.143 | rs12721416 | 0.428 | rs11168307 | 0.773 |
| rs58379944 | 0.144 | rs2228572 | 0.431 | rs2107301 | 0.778 |
| rs7965281 | 0.158 | rs58789572 | 0.438 | rs11168263 | 0.780 |
| rs11168280 | 0.177 | rs7965943 | 0.444 | rs11574100 | 0.787 |
| rs4442605 | 0.180 | rs11168306 | 0.449 | rs7974708 | 0.796 |
| rs2525043 | 0.183 | rs11574050 | 0.455 | rs10747524 | 0.798 |
| rs757555 | 0.183 | rs7305032 | 0.465 | rs7311030 | 0.799 |
| rs1859281 | 0.186 | rs12314197 | 0.471 | rs2544037 | 0.802 |
| rs4237856 | 0.216 | rs7965274 | 0.475 | rs2239182 | 0.819 |
| rs4393380 | 0.221 | rs11574053 | 0.483 | rs2544039 | 0.834 |
| rs11574044 | 0.222 | rs73109883 | 0.506 | rs10783221 | 0.839 |
| rs2525045 | 0.225 | rs10459227 | 0.515 | rs12721397 | 0.847 |
| rs886441 | 0.241 | rs7970376 | 0.517 | rs12303561 | 0.848 |
| rs2238136 | 0.243 | rs11168325 | 0.538 | rs10459217 | 0.850 |
| rs4254129 | 0.246 | rs7967673 | 0.542 | rs4760648 | 0.856 |
| rs11574041 | 0.252 | rs11574081 | 0.548 | rs12299534 | 0.863 |
| rs7975128 | 0.255 | rs731236 | 0.549 | rs11168261 | 0.877 |
| rs12721370 | 0.270 | rs61553170 | 0.555 | rs61558228 | 0.882 |
| rs11574005 | 0.273 | rs12321826 | 0.558 | rs74085273 | 0.898 |
| rs2238140 | 0.278 | rs11168314 | 0.563 | rs11168264 | 0.910 |
| rs2853560 | 0.291 | rs4341603 | 0.565 | rs2239186 | 0.925 |
| rs4307774 | 0.292 | rs2238138 | 0.577 | rs2189480 | 0.934 |
| rs11168311 | 0.308 | rs2408876 | 0.580 | rs11168277 | 0.953 |
| rs4760674 | 0.331 | rs2544038 | 0.580 | rs3890734 | 0.954 |
| rs4328263 | 0.334 | rs2853561 | 0.619 | rs4760658 | 0.954 |

1. Wald test p-value from Cox Proportional regression models adjusted for age, sex, smoking status, alcohol, AFB smear status, HIV status, and cavitary disease; SNPs modelled additively.
